# B Cells and Microbiota in Autoimmunity

**DOI:** 10.3390/ijms22094846

**Published:** 2021-05-03

**Authors:** María Botía-Sánchez, Marta E. Alarcón-Riquelme, Georgina Galicia

**Affiliations:** 1 GENYO, Center for Genomics and Oncological Research, Pfizer-University of Granada-Andalusian Regional Government, 18006 Granada, Spain; maria.botia@genyo.es (M.B.-S.); marta.alarcon@genyo.es (M.E.A.-R.); 2 Unit of Chronic Inflammatory Diseases, Institute of Environmental Medicine, Karolinska Institutet, 17177 Solna, Sweden

**Keywords:** microbiota, dysbiosis, autoimmunity, GALT, Peyer’s patches, inflammation, IgA, B cells, MALT

## Abstract

Trillions of microorganisms inhabit the mucosal membranes maintaining a symbiotic relationship with the host’s immune system. B cells are key players in this relationship because activated and differentiated B cells produce secretory immunoglobulin A (sIgA), which binds commensals to preserve a healthy microbial ecosystem. Mounting evidence shows that changes in the function and composition of the gut microbiota are associated with several autoimmune diseases suggesting that an imbalanced or dysbiotic microbiota contributes to autoimmune inflammation. Bacteria within the gut mucosa may modulate autoimmune inflammation through different mechanisms from commensals ability to induce B-cell clones that cross-react with host antigens or through regulation of B-cell subsets’ capacity to produce cytokines. Commensal signals in the gut instigate the differentiation of IL-10 producing B cells and IL-10 producing IgA+ plasma cells that recirculate and exert regulatory functions. While the origin of the dysbiosis in autoimmunity is unclear, compelling evidence shows that specific species have a remarkable influence in shaping the inflammatory immune response. Further insight is necessary to dissect the complex interaction between microorganisms, genes, and the immune system. In this review, we will discuss the bidirectional interaction between commensals and B-cell responses in the context of autoimmune inflammation.

## 1. Introduction

B cells play a pivotal role in the dialogue between mucosal microbiota and the host´s immune system due to their capacity to differentiate into antibody-secreting cells that produce a substantial amount of IgA antibodies that are released within the mucosa. IgA dimers and J-chain antibody complexes produced in the lamina propria are transported through the epithelial cells after binding the poly-Ig-receptor (pIgR) on the basolateral side of the epithelial cells [1]. The IgA B-cell response is fundamental to maintain a healthy microbiota ecosystem; therefore, any modification in the IgA response will impact the microbial abundance and diversity in the gut mucosa leading to dysbiosis. This dysbiosis may lead to autoimmune inflammation under the proper conditions [2]. At the same time, gut commensals determine the type of IgA response: pathobionts of commensals with invasive properties induce T cell-dependent IgA responses, while the vast majority of bacteria induce T cell-independent IgA responses [3]. The origin of autoimmunity has long been suggested to be the cross-recognition of antigens on microorganisms, whether bacteria or viruses, and host antigens by molecular mimicry. Certainly, some mimotopes have been found in commensal bacteria that have been suggested to participate in the induction of autoimmune pathogenesis [4,5], as well as the production of antibodies to host antigens [6]. Apart from the local humoral response in the gut mucosa, the passage of bacterial components and whole bacteria, even in the steady state, provokes a systemic antibody response beyond the gut mucosa that results in circulating antibodies to gut resident bacteria, which increases in case of inflammation as the intestinal barrier becomes more permeable [7]. Besides producing antibodies, B cells can also serve as antigen-presenting cells (APC) and can produce substantial amounts of cytokines. IL-10 producing B cells, also known as regulatory B cells (Bregs), have the capacity to suppress autoimmune inflammation. The induction and differentiation of Bregs depend on signaling through innate immune receptors. Thus, it is not surprising that gut resident bacteria or bacterial metabolites are able to induce abundant production of IL-10 in B cells with anti-inflammatory activity in autoimmunity [8]. Moreover, bacterial metabolites can also suppress the production of inflammatory cytokines by B cells [9]. Although increased insight into the complex crosstalk between microbiota and immune response mediated by B cells in autoimmunity has been gained, we still need to understand fundamental questions. Is gut dysbiosis a bystander result of inflammation, or is it one of the environmental instigators of autoimmune inflammation in susceptible hosts? Do genetic traits define the gut microbiota composition, either by regulating the IgA response or by regulating the function of epithelial or immune cell subsets? This review reports our current understanding of how the B cells regulate and responds to the microbiota composition in autoimmunity.

## 2. B-Cell Lymphopoiesis and B-Cell Differentiation

The rising of B cells, a branch of lymphopoiesis, takes place in the bone marrow, a specialized primary lymphoid tissue. The cellular composition and specialized microenvironment of this organ allow the common lymphoid progenitor cell to give rise to B cells, ready to encounter their antigen. This possibility is given by a net of specialized non-lymphoid stromal cells and the surrounding osteoblasts located in intimate contact with the B-cell precursors and the to be, B cells [10,11,12]. As a consequence of this organization, the contribution supplied by stromal cells and osteoblasts is two-fold. First, establish adhesion contacts with the developing lymphocytes through cell-adhesion molecules and their ligands. Second, secrete cytokines and chemokines that can control differentiation and proliferation processes [13,14].

In this context, to achieve the stage of the common lymphoid progenitor from stem cells, it is mandatory that the expression of the cell-surface receptor tyrosine kinase FLT3 and the transcription factor PU.1, together, induce the expression of the IL-7 receptor [15,16,17]. For the next step, osteoblasts and, to a lesser extent, stromal cells, produce IL-7, a cytokine essential for the growth and survival of developing B cells in mice. This necessary role has been exposed in experiments using mice that lack either IL-7, its receptor (IL-7R), or any of the molecules implicated in the IL-7R signaling cascade, where all tested animals exhibited a severe block in B-cell development [14,18].

The commitment of the B lineage is achieved along this process and is then when the early pro-B stage is defined. If this state is successfully accomplished, the genes Rag1 and Rag2 are expressed, initiating V(D)J recombination assembly [19,20,21,22], starting Ig heavy-chain rearrangement. This, together with the expression of the transcription factor Pax5, the surface molecule CD19, and the rearrangement of the Ig light chain, gives place to the late appearance of the pro-B cells [23,24]. When the µ heavy-chain locus is fully synthesized, the pre-B-cell receptor (pre-BCR) is complete, and the cell overtakes a tolerance checkpoint rendering it not to recognize self-structures, entering the pre-B state. This change carries an attenuation in IL-7R signaling in order to allow the cell to evolve into further stages [25,26]. These cells will be able to emerge from the bone marrow into the periphery once the pre-BCR couples with the rearranged light chain, allowing the IgM to be expressed on the cell surface (and, if needed, to be secreted as soluble IgM, sIgM) and becoming hence an immature B cell characterized by the expression of high levels of sIgM and low IgD [27,28]. When B cells enter the periphery, they undergo a final maturation step, when they encounter peripheral self-antigens and undergo tolerance creating a repertoire of B cells whose BCR activates following the recognition of foreign antigens.

It is known that B-cell development taking place during and after late fetal life is different from that occurring early in fetal ontogeny [29,30]. The process maintains the same general structure and stages of B-cell differentiation, but cells that develop from these early fetal progenitors give rise to lymphocytes populating mucosal and epithelial tissues involved in innate immune responses. In the adult lymphoid tissues, this population, known as B-1 B cells, represents a minor percentage in secondary lymphoid organs [29,31,32]. Up to this point, differentiation is antigen-independent [33,34].

The small intestine lamina propria (SILP) is, besides the bone marrow, a site for BCR diversification and B-cell development. Particularly during a small window at mice’s early life, RAG2-expressing pro- and pre-B cells undergo VDJ recombination in the SILP, and immature B cells undergo BCR editing [35]. RAG2+ B cells increase after colonization by gut commensals. Developing B cells expressing the TDT enzyme, important in the addition of nucleotides in the VH genes, localize mainly opposite to the luminal surface. In addition, the VH repertoire was found to be similar in BM and SILP developing B cells, but the V repertoire differed, possibly influenced by components of the commensal microbes, because microbial colonization exclusively increased the expression of immunoglobulin kappa light chain in SILP B cells.

**Figure 1:**
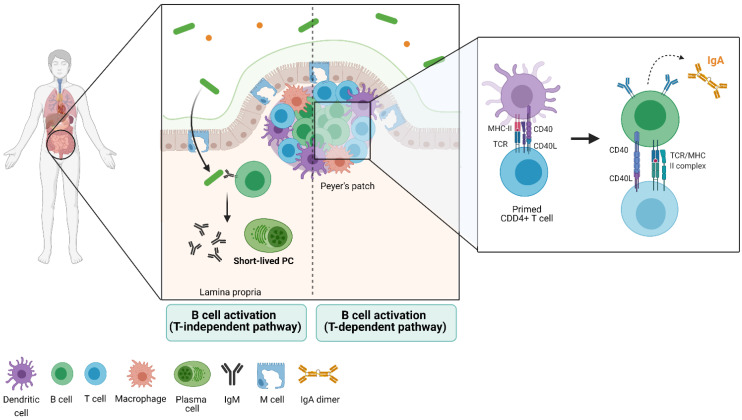
B-cell activation and response in the gut shape the gut microbiota through IgA secretion. When naïve B cells reach the mature state, they can activate and differentiate toward plasma cells (PC) through two pathways in the gut. B cells can encounter antigens in extrafollicular areas within the lamina propria, a process that induces their transformation into IgM-secreting PC. This pathway constitutes the T-independent activation of B cells. This population is characterized by low expression of the activation-induced deaminase (AICDA or AID), the reason why they do not undergo somatic hypermutation nor class switch recombination. Alternatively, B cells can enter the follicular areas located in the Peyer’s patches, where they closely interact with previously primed CD4+ T cells. These lymphocytes have already been in contact with dendritic cells and have experienced antigen presentation, allowing the T cells to activate B cells. This T-dependent activation induces proliferation of B cells, giving place to germinal centers (GCs) and expression of AID and BCL6. Together, these factors allow activated B cells to undergo somatic hypermutation, class switch recombination, and ultimately, IgA synthesis. Newly-secreted IgA binds the local bacteria and shapes the microbiota composition. This image has been created with Biorender.

To reach this stage, B cells need to activate, entering the antigen-dependent recognition phase [36]. To achieve this, B cells follow two paths: 1) the encounter of a T-independent antigen in the extrafollicular areas of the lymph nodes and spleen, a process that induces maturation of B cells and their transformation into IgM-secreting plasma cells. This subset of B cells is mainly characterized by low expression of the activation-induced cytidine deaminase (AICDA or AID), giving place to plasma cells with little class switch recombination [37,38] (Figure 1).

In the second path, naïve B cells can enter the follicular areas and closely interact with antigen-presenting T cells, where they activate in a T-dependent manner, proliferating and creating germinal centers (GCs) [36,39,40]. Within these structures, B cells express BCL6 (transcription factor exclusive of this stage) and AID, which lead to the formation of plasma cells that undergo somatic hypermutation (SH) and class switch recombination (CSR) [36,41,42]. The clonal selection that takes place in GCs has been widely studied, as they usually form transiently in lymphoid organs in response to infection and immunization (Figure 1).

In lymphoid organs associated with the gut, however, germinal centers are invariably present [43,44]. In this context, the differentiation into memory B cells is primarily generated through T-independent responses [45]. Overall, IgM-producing plasma cells (PCs) may tend to live a few days in mice and humans (short-lived PC) or up to a year in mice and decades in humans (long-lived PC) [46,47,48]. The process to a PC state is mainly determined by the repression of the transcription factors that establish B-cell identity during its development, Pax5 and BCL6 [23,24,49]. The expression of Blimp-1, a transcriptional repressor, is required to achieve full PC differentiation. In mice deficient for the gene encoding Blimp-1, *Prdm1^−/−^*, early pre-plasmablasts are still present [23,50].

Only a small percentage of immature B cells leaving the bone marrow and entering systemic circulation become fully mature B cells. This selection process is driven by the limited number of lymphoid follicles in which mature B cells can locate, become activated, and proliferate [47,51,52,53]. These survival niches for PCs are located in the bone marrow, gut-associated lymphoid tissues (GALT), lymph nodes, and spleen [53,54,55,56].

## 3. Gut Immune Structure and Immune Cell Distribution (the Gut-Associated Lymphoid Tissue)

The immune function carried out in the gut involves a group of organs and structures that together form the gut-associated lymphoid tissue (GALT). The GALT, jointly with the draining mesenteric and caudal lymph nodes, coordinates B-cell function. These secondary lymphoid tissues include Peyer’s patches (PP), isolated lymphoid follicles (ILF), the appendix, and, subject to controversy, tonsils and adenoids in the throat [57,58]. Peyer’s patches constitute important sites for the initiation of the immune response in the gut, organized in visible structures composed of lymphocytes projecting into the lumen. These structures are much richer in B cells than peripheral lymphoid organs, as they contain a large number of follicles with small T cell areas and germinal centers in which B cells differentiate and proliferate. PPs are a major IgA-induction site [59,60] and are located primarily along the small intestine. In the large intestine, similar structures have been described and termed lymphoid tissue resembling Peyer’s patches [61]. The presence of PPs during fetal development has been recently shown, although these structures do not develop completely until birth [62]. In parallel, ILFs locate all over the lamina propria, being abundant in the large intestine, being different from PPs in their structure and size, and not visible macroscopically. The amount of ILFs correlates with the higher load of microorganisms as these lie closer to the caecum [63].

ILFs host few GCs, and only with bacterial overgrowth may develop important GC reactions. A greater percentage of B cells in this area are thought to develop after birth in response to antigenic stimulation that occurs as a consequence of the colonization by commensal microorganisms. A layer of epithelial cells and specialized epithelial cells, namely enterocytes and microfold cells (M cells), respectively, is covering these structures and separating them from the lumen. M cells have a luminal surface with fenestrations instead of microvilli and lack the ability to secrete enzymes or mucins, exposing them directly to microorganisms. Consequently, these cells are the preferred route for antigens to enter the PPs [63,64].

The structure of the GALT allows the continuous presentation of antigens from the gut lumen, mainly through M cells by endocytosis or phagocytosis. For several bacterial genres, this process is mediated recognition of specific proteins (e.g., FimH protein present in bacteria and its interaction with GP2 in the M cell surface). The transport following endocytosis and the secretion from the lumen to the basal membrane constitute transcytosis and is often triggered by specific transport systems, such as those present in bacteria belonging to the genera Salmonella and Yersinia [65,66]. In contrast, bacteria without these mechanisms are unable to enter the PPs through the M cells and are thus unable to trigger an IgA response. In addition to this model, other mechanisms have been suggested, such as the one followed by commensal bacteria to cross the epithelium to be phagocytosed by macrophages and dendritic cells. Among these, transport through the gaps between epithelial cells allows antigens to directly access the PPs. One way or another, active uptake of antigens by M cells stimulates the expression of CCL20 and CCL9 by epithelial cells, which by gradient attract DCs to the area through interaction with CCR6 and CCR1, respectively. DCs are then responsible for T lymphocyte activation, which, in turn, induce naïve B cells to isotype switching toward IgA [67,68]. The cecum may also play an important role in the regulation of IgA production in the colon, but details are still largely unknown.

The importance of CSR toward IgA lies in the wide variety of roles this immunoglobulin can play. On the one hand, IgA has the ability to neutralize bacteria and viruses in general as well as to bind pathogenic microbes and toxins with high affinity, in particular. This process facilitates their elimination. This binding, however, is not exclusive of pathogens, as IgA can also bind commensal microbiota with low affinity, contributing to the regulation of commensal composition. The binding of IgA to bacteria fosters their internalization into the PPs because IgA can bind M cells and stimulate endocytosis [61,69,70]. On the other hand, IgA possesses a spatial structure that renders it to activate complement, which in turn prevents a pro-inflammatory response occurring in the gut in a steady state. As a consequence of the exposed functions, IgA controls the microbiota composition. Work carried out with mice lacking IgA shows altered microbiota or dysbiosis but also a less diverse microbiota [71].

## 4. The Microbiota

All along the gastrointestinal tract, the epithelium is covered by a layer of mucus mainly composed of gel-forming mucins. The main function of this structure is to provide protection against a collection of viruses, fungus, and bacteria inhabiting the host´s mucosa, collectively known as microbiota. When analyzing bacterial distribution, the abundance is relatively low in the stomach and the duodenum [72], increasing distally. Hence, the ileum contains around 10^8^ bacteria per millimeter of lumen, reaching up to 10^11^ in the colon. These microorganisms live in an ecological relationship named commensalism, where both microorganisms and their host obtain mutual benefits from each other. To illustrate, the host benefits from bacteria since bacteria participate in the degradation of complex food carbohydrates and their transformation into simple sugars [73]. The epithelium of the mucosa and the immune system have the delicate task of maintaining a commensal relationship while at the same time keeping away pathogens from the gut and the system. The main modifiers of the microbiota are food, antibiotics, prebiotics, and probiotics.

## 5. The B-Cell Response to the Microbiota

The mucosal B-cell response is coordinated by Toll-like receptor (TLR) and BCR signaling. As described before, the production of IgA has a major role in the interaction between the immune system and the gut microbiota. The majority of mucosal IgA plasma cells are derived from the B-cell activation occurring in the mucosal-associated lymphoid tissue (MALT), located along the GALT. Even though mesenteric lymph nodes (MLN) are not directly considered GALT, because these are not in direct contact with the lumen, they do receive afferent lymphatics from the gut and lymphocytes that migrate from the PPs. The role of the MLN in IgA B-cell responses is controversial, but due to its role in tolerance induction, the MLN is very important in the maintenance of gut homeostasis. As it occurs in other sites of the immune system in the body, other cells also perform a role in the gut as antigen presenters and as activators of immune responses. Antigen presentation by dendritic cells (DCs) in the GALT can activate T and B cells or migrate to intrafollicular T cell areas in the PPs and prime CD4 T cells forming, together with activated B cells, the GCs in the B-cell follicle. CSR and IgA production are controlled by several factors. For instance, TGF- is important in IgA CSR. Other regulators involved are CD40-CD40L signaling, CD40-TLR signals, as well as retinoic acid-dependent signals.

Recent advances in the study of the B-cell response toward commensal biology show that this response can be elicited both in the gut compartment and systemically [74]. In the gut, associated secondary lymphoid structures, including Peyer´s patches, isolated lymphoid follicles, and mesenteric lymph nodes, there are germinal center niches for activated B cells that undergo somatic hypermutation and affinity maturation. Gut-associated germinal centers (GaGCs), differently from GC transiently formed after immunization or infection, persist chronically because they are constantly exposed to microbes and food antigens that shape the B-cell response in the gut. Peyer´s patch germinal centers (PP-GC) express a set of public clonotype-specific antibodies persistently. These clonotypes are dependent or independent of gut commensals as clonotypes differ between germ-free mice and specific pathogen-free mice, and transfer of fecal commensal from SPF mice to germ-free mice restored commensal-dependent clonotype indicating a concomitant BCR selection. These B-cell clonotypes undergo affinity maturation in PP GCs, generating highly selected antibodies that show increased binding compared to their unmutated precursors to commensal bacteria and could contribute in a steady state to the symbiotic relationship between the host and the microbiome. Antigen-driven selected B-cell clonotypes can aim at specific bacterial populations, and at the same time, could be protective against pathogens through cross-reactivity on the surface glycans of microbiota [75]. Similarly, germinal centers in mesenteric lymph nodes (mLN) undergo clonal selection and antigen-driven maturation that are shaped by the presence and complexity of the gut microbiota composition. Interestingly, reports agree on the observation that B-cell clones in germ-free (GF) mice are enriched in clonotypes similar across mice (public clonotypes) that may be ontogenically defined and BCR-dependent [76]. These clonotypes seem to be replaced by commensal specific clones upon colonization with fecal bacteria or by a consortium of bacteria that shapes the B-cell repertoire. In summary, GF mice have antibodies against microbiota components, and these antibodies are the starting repertoire that supports the construction of the gut IgA repertoire against commensals [44,75].

The mucosal exposure to commensals triggers a B-cell response that mainly results in an IgA response involving a distinctive and restrictive repertoire of memory B cells and plasma cells directed against membrane antigens. Despite encountering high levels of mucosal bacteria and successive bacterial exposures, this repertoire does not undergo diversification through SH. It is possible that IgA mucosal protection may require generic and low-affinity responses that adapt sequentially to many different varieties of overlapping antigen exposures [74]. The functional demand against challenges from the microbiota determines the B-cell response and its extent in the gut mucosa. Of interest, the same B-cell clone is present in multiple PPs, and there is a reuse of germinal centers resulting in highly synchronized, oligoclonal, and affinity matured IgA responses in the gut [74]. Furthermore, in a steady state, some bacteria penetrate mucous membranes and prime in a systemic way the secondary lymphoid structures. On the other hand, the translocation of bacterial particles or whole bacteria from the gut mucosa into the system also triggers the activation of commensal specific B cells. Systemic bacterial exposure generates a more diverse and flexible repertoire dominated by IgG antibodies against the membrane and cytoplasmic bacterial fractions, while gut bacterial exposure induces IgA production that recirculates systemically. Both may serve to prevent sepsis following intestinal disruption [77]. Overall, commensals shape the systemic antibody and B-cell repertoire. A recent report showed that gut-educated IgA+ plasma cells move to the central nervous system meninges, where they secrete anti-microbial antibodies locally to prevent the entrance of microbes into the CNS parenchyma [78] in a homeostatic situation. This phenomenon was more pronounced after a breach of the gut intestinal barrier and after systemic infection with a pathogen with tropism for meninges.

When B cells are stimulated by resident gut commensals, they not only produce antibodies but also produce cytokines. Using a rheumatoid arthritis model, it was shown that gut resident commensals promote the production of IL-10 in both mLN and spleen by inducing the production of IL-6 and IL-1 in macrophages. The IL-10 producing B cells bear a transitional two-marginal zone precursor B-cell phenotype (T2-MZPs) that may be educated in the gut. Most of them express α-4-β-7 integrins, indicating that they can traffic to and from the gut [79]. Furthermore, Mishima et al. [80] demonstrated in vivo that resident microbiota, on the one hand, and bacterial in vitro stimulation on the other induced the production of IL-10 in gut resident B cells from mLN and colon lamina propria. The production of IL-10 depends on the activation of the TLR2/Myd88 and downstream signaling through the PI3K (p110d subunit) pathway. The regulatory phenotype of the gut resident B cells is maintained by continued bacterial stimulation and helps to preserve gut homeostasis by downregulating the production of pro-inflammatory cytokines by the B cells themselves and by T cells [80].

Bacteria can also induce the differentiation of IL-10 producing B cells through the production of acetate, a sub-product of soluble fiber metabolism [81]. Finally, IL-10-producing plasmablasts and plasma cells exert regulatory functions during autoimmune inflammatory processes [82]. Of special interest, IgA+ plasma cells that appear to originate in the gut lamina propria traffic to inflamed sites far from the gut and dampen inflammation [83].

## 6. Shaping the Microbiota Composition by the B-Cell Response

IgA is the most abundant mammalian antibody isotype lining mucosal tissue and the gut mucosa. The IgA response in the GALT involves both T cell-dependent and T cell-independent pathways of differentiation. The T cell-independent response appears to produce polyreactive IgA antibodies that bind commensal bacteria, while the T cell-dependent IgA response is triggered by either enteric pathogens or pathobionts [3,76]. The diversity and abundance of bacterial species are defined by the extent of IgA production within the gut mucosa. The microbiota diversity is determined by the difference in genetic background between different strains, C57BL/6 mice and BALB/c mice that differ in gut polyreactive IgA levels [71]. For instance, BALB/c mice that have an increased number of IgA+ cells in the PPs and the LP as compared to C57BL/6 mice have a more diverse microbiome. Cohousing or heterologous fecal matter transplant between strains did not rescue the microbiome diversity of C57BL/6 mice neither the IgA production. Similarly, mice deficient in activation-induced cytidine deaminase (AID), a master regulator of class switching and somatic hypermutation, produce only unmutated IgM Abs and are devoid of IgA. These mice have a dysbiosis characterized by the overexpansion of segmented filamentous bacteria (SFB). While *AID^G23S^* mice, which carry a mutated form of AID that allows class switching, but not hypermutation of the BCR, produce unmutated IgA and have an expansion of gut microbes distinct from that of wild-type mice [84].

As in mice, human IgA shapes the gut bacterial community. Humans with selective IgA deficiency (SIgAD) exhibit a dysbiosis that consists in the expansion of bacteria with pro-inflammatory capacity and diminution of anti-inflammatory bacterial species, suggesting that IgA has both functions, that of restraining expansion and that of promoting colonization of different bacteria taxa [85]. Importantly, an increased systemic Th17 response was associated with dysbiosis in individuals with SIgAD [86].

## 7. The Interaction Between the Gut Microbiota and B Cells in Autoimmune Diseases

**Figure 2:**
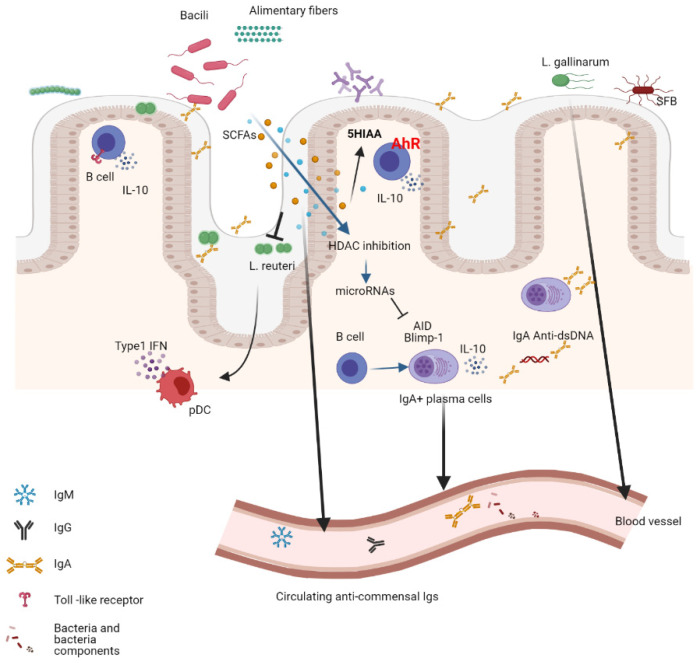
Resident intestinal commensals modulate the local and systemic immune response. Bacterial components trigger the humoral response within the GALT and also systemically when they move into circulation. Similarly, the metabolites produced by bacteria (SCFA) from alimentary fiber exert local and systemic functions reducing inflammation and inducing regulatory B cells in autoimmunity. This image has been created with Biorender.

Autoimmunity is the result of a complex interaction between genetic and environmental factors, most of which are not well defined. However, in the last decade, compelling evidence suggests that mucosa commensals participate in the pathogenesis of autoimmunity. The ways in which microbiota contribute to disease are very diverse. Commensal bacteria can induce regulatory mechanisms to diminish the excessive inflammation associated with autoimmunity, or on the contrary, commensal bacteria can fuel the inflammatory autoimmune response or even contribute to triggering autoimmunity. Here we will review what is known and has been observed in several autoimmune diseases (Figure 2).

### 7.1. Systemic Lupus Erythematosus

In an animal model of lupus, bacterial DNA CpG motifs were potent inducers of IL-10 production in B cells. Bacterial DNA induced regulatory B cells that produced two regulatory cytokines, IL10 and IL35. Depletion of the microbiota with oral vancomycin at early ages led to a more severe disease, while oral delivery of bacterial DNA increased the differentiation of regulatory B cells. Regulatory B cells differentiated In vitro with bacterial DNA could mitigate the severity of the disease [8]. Food is an important modulator of the composition of the gut microbiome. The overexpansion and translocation of *Lactobacillus reuteri*, together with increased gut permeability and disease severity, have been described in a TLR7 mouse model of lupus. The expansion and the *L. reuteri* translocation were inhibited by feeding the lupus mice with dietary-resistant starch. This effect is mediated via the fermentation of dietary fiber into SCFA and is accompanied by a reduction in the production of type I IFN, an important disease modulator, and disease severity [87].

Lupus pathology can also be dampened by SCFAs through direct modulation of the B-cell response. Propionate and butyrate result from gut-microbiota processed dietary fibers. These metabolites mediate the reduction of AID and Blimp-1 expression through the upregulation of miRNAs that target *Aicda* and *Prdm1* 3′UTRs, inhibiting histone deacetylation of the genes. As a consequence, a reduction in CSR, somatic hypermutation, and plasma cell differentiation were observed, impacting the autoimmune response in MRL/Fas*^lpr/lpr^* and NZB/W F1 mice treated with SCFAs. These mice showed reduced IgG1 and IgG2a anti-dsDNA antibodies as well as reduced skin and kidney damage [88].

Other bacteria-associated metabolites that have been implicated in the pathogenesis of lupus are tryptophan and kynurenine. Bacterial taxa developed on a high tryptophan diet in the lupus-prone B6.*Sle1.Sle2.Sle3* mice transferred onto germ-free C57BL/6 mice induced autoantibodies and a lupus-like phenotype, suggesting that dietary tryptophan modulates the development of a pro-inflammatory microbiota [89]. Commensals can contribute to disease pathogenesis through the induction of autoreactive IgA+ B-cell clones capable of secreting anti-dsDNA and anti-nucleohistone antibodies in the gut. The production of these IgA autoantibodies in the gut correlates with the severity of the inflammation and the onset of proteinuria characteristic of the kidney disease in lupus. IgA autoantibody levels in lupus mice are reduced through the depletion of the microbiota with antibiotics [6].

It has long been proposed that antigen mimicry could be a mechanism of autoimmune triggering, being the production of autoantibodies the result of B-cell responses against external antigens that cross-react with self-molecules, and bacterial components may be one of such antigens. It was recently found that the systemic humoral responses against pathogenic bacteria that cause urinary tract infection were associated with lupus flares. Uropathogenic bacteria generate a biofilm containing amyloid curli protein that, when complexed with bacterial DNA, stimulates the production of antibodies directed against DNA and protein, cross-reacting with self-DNA in lupus patients [90]. Similarly, the systemic humoral response in lupus patients against the wall lipoglycans of commensal *Ruminococcus gnavus* was reported to directly correlate with the severity of the disease and with levels of anti-dsDNA antibodies [91]. Furthermore, orthologs of the RNA binding autoantigen Ro60, present not only in lupus but also in Sjögren´s syndrome, were found in specific species of commensal bacteria present in human mucosal tissues. The colonization of germ-free mice with such bacteria triggered an anti-Ro60 B and T cell response and the deposition of immunocomplexes in the glomeruli [4]. Another gut commensal that translocates from the gut mucosa into the system is the *Enteroccoucus gallinarum*, which was found to be capable of inducing a cellular and humoral autoimmune response through the aryl hydrocarbon receptor (AhR)-CYP1A1 pathway triggering Th17 cell activation and anti-dsDNA antibody production in genetically susceptible lupus mice [92]. This collection of evidence suggests that both pathogenic and commensal bacteria may contribute to the pathogenesis of lupus.

### 7.2. Multiple Sclerosis

The hallmark of multiple sclerosis (MS), an autoimmune neuroinflammatory disease, is the presence of autoantibodies against the myelin oligodendrocyte glycoprotein (MOG). However, the stimulus triggering the autoimmune process is not yet known. In general, MS has been commonly associated with environmental factors, in particular with microbial infections [93]. In the last decade, in an attempt to elucidate a possible cause attributable to disease onset, several variables have been analyzed, including the microbiota composition and the B-cell response generated to it. In this line, a two-phase scenario has been defined using a relapsing-remitting (RR) murine model of spontaneous experimental autoimmune encephalomyelitis (EAE). In specific pathogen-free (SPF) environments or germ-free conditions (GF), the animals possess a proper B and T function. By assessing disease development and severity, a first expansion of CNS autoreactive CD4+ T cells in the GALT has been established, followed by the recruitment of autoantibody-producing B cells [94]. This was the first study to provide evidence of activation of MOG-specific T cells in the gut as a necessary but not sufficient trigger for the development of EAE. After this study, a relationship between the gut microbiota and MS has been established, followed by several studies aimed at characterizing the microbiota composition and the products of microbiota metabolism. In this context, short-chain fatty acids (SCFA) have received increased attention in line with the finding of the immunomodulatory effects of these metabolites [93]. The mechanism through which SCFA induces an anti-inflammatory program is based on the increase in regulatory T cells (Treg) and a reduction of pro-inflammatory T cells. In vitro studies with the various bacterial metabolites have shown the ability of these in decreasing the differentiation of Th17 cells, having propionate the most marked effect [95], and in increasing the numbers of FoxP3+ T cells in spleen and lymph nodes [96]. This data, together with an observed low production of IFN-γ suggests a direct anti-inflammatory effect of the SCFA. However, the role that SCFA plays during the onset and disease course of MS has never been directly studied.

Overall, a comparison of the microbiota composition between MS patients and healthy controls shows no major differences in bacterial variety neither in children nor in adults [97,98]. On the other hand, subtle differences have been identified in commensal communities. For instance, species such as *Parabacteroides distasonis,* a species widely known to cause an increase in Treg generation, is reduced in MS patients as compared to healthy controls [99], while other bacteria such as *Akkermansia* or *Methanobrevibacter* are increased in MS patients [100]. In agreement with the EAE data, *Akkermansia muciniphila* has been associated with a strong pro-inflammatory T cell response in vitro [99]. However, there are some genres that cause controversy, as in the case of *Prevotella*. Within this genre, the effects may differ according to the species. For example, *P. histicola* has been shown to prevent disease activity in mice [101], but the relative abundance of this genre is reduced in patients with the highest clinical scores [102]. Summarizing the various experimental studies in MS, particularly the relapsing-remitting type of disease, patients have gut dysbiosis, which could be the cause of the lower amount of bacterial species during MS attacks. Consequently, this would suggest that microbiota composition might play an active pro-inflammatory role in the development and/or exacerbation of MS. As further evidence, mice with induced EAE transplanted with stool from MS patients suffered an exacerbation of neurological symptoms [99,103].

Understanding the established relationship between the immune system and the microbiota composition in the GALT as tight and mutually dependent, several studies have attempted to elucidate the cellular mechanisms behind the effect of microbiota in autoimmunity. In MS, dysbiosis induced by the administration of antibiotics ameliorated the disease as assessed by motor dysfunction, axon damage, and T cell infiltration in the CNS. Notwithstanding, re-colonization of the commensal microbiota not only worsened the disease but also decreased the number of FoxP3+ CD39+ T cells [104]. This observation supports the role of CD39+ T cells in preventing the development of MS [105,106,107], which is supported by evidence showing CD39 as one of the master regulators of Treg cells induced by the host microbiome [108].

Regulatory B cells, as defined by the expression of CD5 and high levels of CD1, as well as by the secretion of IL-10, have also been closely related to MS progression. Soon after their discovery, these cells were shown to be able to dampen the initiation of EAE [109]. Following the administration of antibiotics of broad spectra to mice with EAE, there an increase in Breg numbers in peripheral lymphoid sites was observed [110]. This goes hand in hand with a rise in Breg counts in the spleen and CNS and an increase in IL-10 concentrations in the spinal cord [104]. When taken together, these results seem to show a relationship between the amount of Breg in the CNS, the concentration of IL-10, and an improvement in EAE, but whether this scenario provides a protective effect remains unknown. It was later identified using molecules migration cluster analysis that a small subset of plasma cells (PC) located in the CNS of EAE mice originated in the GALT and possessed the ability to produce IgA. This observation, together with the dramatic reduction of PCs observed in the gut of these mice during relapses, indicates a mechanism of migration of the PCs to the periphery and the possibility of having a specific role in EAE progression. This was confirmed by the fact that EAE markedly worsened after the removal of PCs and plasmablasts (PB), and the disease strikingly ameliorated following the external administration of gut-derived IgA+ PC [83].

A greater diversity of IgA-bound bacteria in EAE mice was observed when analyzing the taxa stimulating the CNS-reactive IgA response, with a tendency indicating that OTUs that stimulate an IgA response the most are not necessarily the most abundant within the fecal content [111]. To note, species that display greater levels of IgA binding in EAE mice include *A. municiphila*, *Eggerthella lenta*, and *Bifidobacterium adolescentis*. Confirmation of an IgA-producing commensal-reactive PC-activated trafficking to the CNS during EAE relapses points toward this cell subset as a new source of IL-10+ Bregs worth to be explored.

### 7.3. Rheumatoid Arthritis

Rheumatoid arthritis (RA) stands for an autoimmune disease caused by abnormal immune triggering that breaks tolerance against synovial antigens. RA is a chronic pathology characterized by synovial inflammation, joint pain, cartilage destruction, and bone erosion and damage-causing important deformation [112,113]. In RA, many studies have been carried out in an attempt to find the initial event that triggers immune dysregulation. Breg cells are one of the most recently studied candidates, as they are a pivotal B-cell subpopulation and can be differentially induced by bacterial microbiota [114]. In this work, the authors have shown in splenocytes how differentiation of naïve B cells toward Bregs (B10, T2-MZP, and Tim-1+ B cells) reaches its peak following the in vitro stimulation with *Escherichia coli* (with strong immunogenicity) when compared to *Bacteroides vulgatus* (with weak immunogenicity). The numbers of Bregs were accompanied by high levels of expression of IL-10 and the pronounced expression of suppressive molecules on the B-cell surface. Bregs had the functional capability to inhibit the proliferation of dendritic cells, prevent the proliferation of Th1 and Th17 cells, and promote Th2 differentiation in vitro. In summary, this study provided for the first time evidence of the ability of gut bacteria to counter-regulate the immune response in a healthy host in order to maintain immune homeostasis.

Several studies tried to correlate RA severity with microbiota composition. In this line, *Fusicatenibacter saccharivorans*, *Dialister invisus*, *Clostridium leptum*, *Ruthenibacterium lactatiformans*, *Anaerotruncus colihominis*, *Bacteroides faecichinchillae*, *Harryflintia acetispora*, *Bacteroides acidifaciens*, and *Christensenella minuta* are species shown to be reduced in RA patients, although their individual contribution remains unknown [115]. Specific species and genus analyses in humans include a report on the abundance of *Collinsella* in RA patients correlating with IL-17A levels [116]. The reason why the expansion of this bacteria species is believed to induce a pro-inflammatory environment due to a loss of gut epithelium integrity is that in vitro, *Collinsella* has been shown to increase gut permeability in human cells through the downregulation of tight epithelial junctions. In addition, increased abundance in the early stages of RA of the *Prevotellaceae* family has been reported [117,118].

It is widely accepted that RA is not a one-manifestation disease. Instead, RA patients tend to present different profiles. Studies classifying patients for their IgA and IgG levels show that the predominance of one or another can be associated with the relative differential abundance of *Prevotella copri* [119]. However, although it is clear that this microorganism must play some role in disease development, the nature of this role remains unclear. Fecal analyses of microbiota composition have clearly elucidated that *Lactobacillus* and those bacteria belonging to the genus *Verrumicrobiae* and *Akkermansia* are significantly increased in RA patients as compared to healthy controls [120]. Notably, similar analyses have found a positive correlation of serum levels of IL-17A and/or TNF-α with the amount of *Enterobacteriaceae* and *Klebsiella* and a negative correlation with the proportion of *Bifidobacterium* and *Haemophilus* spp. [121,122]. The K/BxN model of spontaneous arthritis is mediated by T cells that express both the T cell receptor (TCR) transgene KRN and the MHC class II molecule A(g7) and by autoantibodies recognizing glucose-6-phosphate isomerase (GPI). Remarkably, using this model, Morton et al. demonstrated that Th17 cells that differentiated in the gut trafficked to the spleen from the descending colon. Further, the accumulation of gut-derived Th17 cells directly correlated with titers of GPI antibodies in the serum [123]. In addition, segmented filamentous bacteria (SFB), a gut pathobiont and a powerful inducer of Th17 cells in the PPs has been associated with autoimmune arthritis [124]. The fact that immune cells recirculate from and to the gut confirms the link between gut pathobionts and the immune response at extra-intestinal tissues in genetically susceptible hosts.

The mechanism best characterized in relation to the interaction between Bregs and microbiota is the one mediated by IL-10 secretion. A recent study has brought some light to a possible process. By measuring the levels of PD-L1 in different subsets of Bregs, that is, CD19+ PD-L1+ B cells, CD24^hi^ CD38- PD-L1+ B cells, and CD24^hi^ CD38^hi^ PD-L1+ B cells, the authors noted that all cells having the highest levels of PD-L1 were significantly reduced in rheumatoid arthritis patients when compared to the healthy controls. All cell subpopulations increased in good responders upon treatment. Moreover, the authors show how TLR9 stimulation in vitro induced PD-L1 expression on Breg subsets and that the resulting populations had the ability to functionally suppress T cell responses via PD-1 (CD4+ and CD8+) [125].

Finally, the role that microbiota-derived metabolites play in modulating the suppressive function of Bregs remains poorly understood. Interestingly, upon supplementation with butyrate in collagen-induced arthritis (CIA) mice, amelioration of the joint inflammation mediated by AhR stimulation was observed. An increase in the AhR-dependent gene transcription pathway in the Breg subset CD19+ CD21^hi^ CD24^hi^ is the proposed mechanism [126,127]. This increased transcription, in turn, inhibits GC B cells and PC differentiation [128]. Altogether, these effects would have as a consequence a reduction in autoantibody-producing PCs and hence, a reduced immune activation [129].

### 7.4. Type I Diabetes

Type 1 Diabetes (T1D) is an inflammatory autoimmune disease that is the result of T lymphocyte-mediated destruction of the pancreatic Langerhans cells. It has been until quite recently that the role of B cells has been shown in the pathogenesis of T1D. The early appearance of anti-cell antibodies has been observed in susceptible individuals [130]. In the non-obese diabetic (NOD) mouse model, the lack of B cells [131] or targeting B cells with a B-cell depleting treatment prevents the development of diabetes [132]. As in other autoimmune pathologies, the gut microbiota plays a fundamental role in T1D pathogenesis. Significant changes in the composition of the gut microbiome have been reported in subjects with clinical or preclinical T1D as well as in animal models of the disease [133]. The presence of gut dysbiosis in T1D has been associated with increased gut bacteria bound to IgA and the concomitant reduction in the production of SCFAs. In fact, the levels of circulating IgA were negatively correlated with fecal acetate levels. Transplant of fecal matter from T1D patients into GF-NOD mice resulted in increased gut permeability, higher proportions of IgA-bound gut bacteria, and lower levels of free fecal IgA. In addition, changes in draining LN of the frequency of transitional and marginal zone B cells, and the increased frequency of CD4+IFN+ and CD8+TNF+ T cells, were also observed. While short and long-term treatment with acetate modifies the immune response in and beyond the gut, a diminished frequency of IgA-bound bacteria, free IgA in the cecum, reduction of the frequency of IgA+ B cells in the spleen and draining LNs and PPs, and a reduction of GC B cells in the mLN and PPs, was observed. In addition, diminished gut permeability and induced changes in the gut microbiome composition, and an overall reduction of insulinitis was observed following acetate treatment. The effects of acetate treatment had a direct impact on the function of B cells on cytokine production and on the expression of several genes involved in B-cell differentiation [9]. Moreover, the analysis of the humoral systemic immune response against the gut bacteria of pediatric patients with T1D showed that the IgA response to *A. colihominis*, *B. animalis*, *B. fragilis*, *B. vulgatus*, and *C. perfringens* was lower compared to healthy age-matched controls. A prospective study found that the future diagnosis of T1D was associated with the presence of high-risk HLA haplotypes *DR3* and *DR4* and IgG2 responses against *R. faecis.* This study also revealed an association of the IgG isotype antibodies reactive against gut bacteria, the specificity of the islet autoantibody, and the HLA [134]. Although it is not yet defined whether gut dysbiosis associated with T1D is causal or a consequence of the disease, changes in the gut microbiota appear before the onset of T1D, and interventional studies in the mouse indicate that correction of the dysbiosis prevents the development of the disease. While in humans the evidence is not as robust, gut microbiota maturation products such as breastfeeding, consumption of probiotics, and SCFA seem to have protective effects [133].

## 8. Conclusions and Future Perspectives

The genetic background of the host shapes the gut microbiome, which may affect the incidence of immune-mediated diseases in genetically susceptible individuals. The adaptive immune response is crucial in maintaining the homeostasis of the gut microbiota and its composition. Specifically, B cells that produce IgA have a key role in keeping homeostasis in the gut ecosystem. Thus, any change in the IgA response induced by microbial components may alter the delicate commensal relationship with the microbiota. Moreover, the crosstalk networks sustained between the host and its microbiota are extensive and can be local within the mucosa and between microbiota and various organs of the host. This communication is mediated by molecules and cells. Consequently, dysbiosis may lead to a loss of self-tolerance and propagation of pro-inflammatory signals and the differentiation of effectors cells. On the other hand, modulation of the intestinal microbiota may prevent the immune-mediated disease process. Experimental interventions aimed to amend the dysbiosis associated with autoimmune inflammation could be a promising therapy to prevent or reduce autoimmune progression. Those interventions may include functional foods and/or the introduction of specific bacteria, but as the microbiota is an ecologic niche with multiple interactions among its members, it may be more adequate to introduce a consortium of bacteria, or in particular cases, perform total fecal transplantation.

Much is still needed to understand the gut microbiota-B-cell interaction and the genes involved.

## Data Availability

Not applicable.
